# *Blastomyces gilchristii* as Cause of Fatal Acute Respiratory Distress Syndrome

**DOI:** 10.3201/eid2202.151183

**Published:** 2016-02

**Authors:** Daniel Dalcin, Aaron Rothstein, Joanna Spinato, Nicholas Escott, Julianne V. Kus

**Affiliations:** Northern Ontario School of Medicine, Thunder Bay, Ontario, Canada (D. Dalcin, A. Rothstein, N. Escott);; Public Health Ontario, Toronto, Ontario, Canada (J. Spinato, J.V. Kus);; Thunder Bay Regional Health Sciences Centre, Thunder Bay (N. Escott);; University of Toronto, Toronto (J.V. Kus)

**Keywords:** acute respiratory distress syndrome, Blastomyces dermatitidis, Blastomyces gilchristii, fungi, blastomycosis, respiratory infections, virulence

## Abstract

Since the 2013 description of *Blastomyces gilchristii,* research describing the virulence or clinical outcome of *B. gilchristii* infection has been lacking. We report molecular evidence of *B. gilchristii* as an etiologic agent of fatal acute respiratory distress syndrome. *B. gilchristii* infection was confirmed by PCR and sequence analysis.

Differences in virulence have long been observed between different strains of *Blastomyces dermatitidis* (*1*,*2*). Following the 2013 description of *B. gilchristii* as a cryptic species of *Blastomyces* spp., *B. dermatitidis* and *B. gilchristii* have been recognized as etiologic agents of blastomycosis (*3*). However, research evaluating the differences in clinical outcome between *B. dermatitidis* and *B. gilchristii* infection has been lacking. Here we demonstrate that *B. gilchristii* infection can cause fatal acute respiratory distress syndrome (ARDS) in humans.

## The Study

A 27-year-old woman sought care at an emergency department in a rural community in northwestern Ontario, Canada, for a nonproductive cough, right-sided chest heaviness, nausea, and abdominal pain that had developed over the previous week. She had a previous diagnosis of type 1 diabetes mellitus and a history of intravenous drug use.

**Figure 1:**
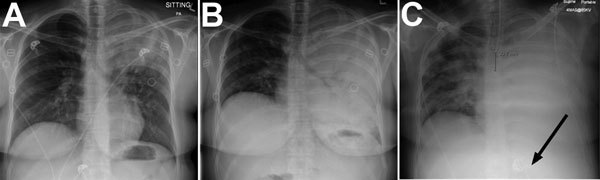
Chest radiograph at various stages of *Blastomyces gilchristii* infection in a 27-year-old woman, Ontario, Canada. A) Day 0: posterior–anterior (PA) chest radiograph at initial emergency department examination. Discrete confluent left upper lobe consolidation with air bronchograms are visible. B) Day 5, 15:10: PA chest radiograph demonstrating complete confluent opacification of the left hemithorax with extensive air bronchograms. C) Day 6, 23:30: PA chest radiograph postintubation with confluent left lung consolidation and new right patchy airspace opacification. Arrow indicates the correct placement of a nasogastric tube.

On examination, deep and labored breathing was observed, and decreased air entry in the left upper lung was heard on auscultation. A chest radiograph revealed left upper lobe consolidation (Figure 1, panel A). Blood chemical analysis revealed diabetic ketoacidosis (25.4 mmol/L glucose, 3.2 mmol/L K+, 6.0 mmol/L HCO_3_−, anion gap 27 mg/dL, and pH 7.0 venous blood gas). Intravenous fluoroquinolone was administered for suspected left upper lobe bacterial pneumonia, and fluid resuscitation and intravenous insulin were administered for diabetic ketoacidosis.

During the first 2 days in hospital, the patient was stable and afebrile, despite remaining tachycardic and tachypneic. From day 3 to day 4, the patient became febrile (38.3°C) and tachypnea worsened to the point that >4 L of O_2_ was required to maintain O_2_ saturation >90%. The patient’s leukocyte count was elevated at 13.5 × 10^9^ cells/L.

On day 5, the patient was observed to be using accessory muscles of respiration. A chest radiograph was performed and showed a complete whiteout of the left lung (Figure 1, panel B). Air transfer to a tertiary hospital was requested but unavailable. The patient’s mean arterial blood pressure decreased from 80 to 65 mm Hg, leukocyte count increased to 18.8 × 10^9^ cells/L, and hemoglobin decreased from 140 to 102 g/L. A chest radiograph revealed patchy opacification of the right lung that was not observed several hours earlier (Figure 1, panel C). After intubation and sedation, the patient was successfully transferred to the intensive care unit of a tertiary hospital.

**Figure 2:**
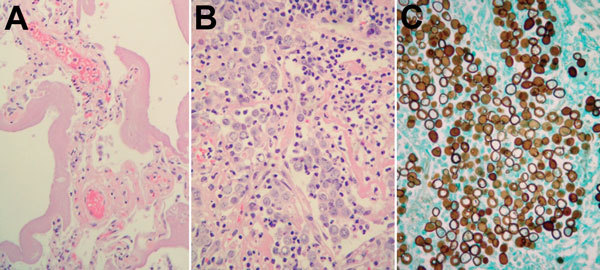
Histologic manifestations of *Blastomyces gilchristii* infection in a 27-year-old woman, Ontario, Canada. A) Nonconsolidated lung. Thick hyaline membranes, characteristic of diffuse alveolar damage and acute respiratory distress syndrome, line the alveoli. Hematoxylin and eosin (H&E) stain, original magnification ×400. B) Consolidated lung. Alveoli are completely filled with *B. gilchristii* yeast cells and neutrophils. *B. gilchristii* cells are pale bluish-gray with a distinct cell border. H&E stain, original magnification ×100. C) Consolidated lung containing abundant *B. gilchristii* yeast cells with characteristic broad-based buds. Grocott methenamine silver stain, original magnification ×400.

On arrival at the intensive care unit, the patient was put on a mechanical ventilator requiring positive-end expiratory pressure to oxygenate. Antimicrobial drugs (vancomycin, piperacillin/tazobactam, and azithromycin), an antifungal drug (amphotericin B), and a vasopressor (norepinephrine) were administered, and sputum cultures were sent for bacterial and fungal culture. One day after entering the intensive care unit, the patient became bradycardic and went into cardiac arrest. Cause of death was determined to be ARDS (Figure 2).

A sputum specimen was prepared for direct microscopic examination for fungal elements by staining with Calcofluor White (Sigma-Aldrich, St. Louis, MO, USA) and for fungal culture by plating onto BHICCGE (brain heart infusion + 5% sheep blood + 50 μg/mL chloramphenicol, 0.01% cycloheximide, 20 μg/mL gentamicin, and egg white) agar and inhibitory mold agar plates and incubated at 28°C. Direct microscopic examination of the sputum specimen revealed broad-based budding yeast with refractile cell walls characteristic of *Blastomyces*. After appropriate culture and incubation, small colonies of mold were observed after 1 week. The identification of a *Blastomyces* sp. was confirmed with a molecular probe (AccuProbe *Blastomyces dermatitidis* Culture Identification Test; Gen-Probe, San Diego, CA, USA) and by conversion of the mold-form to the yeast-phase using Blasto-D medium and incubation at 37°C (*4*). The culture was preserved at −80°C as a glycerol stock with the designation 13BL347.

The isolate was later resurrected and incubated as previously described (*4*). Using a similar method described in Brown et al. (*3*), fungal DNA was extracted, the internal transcribed spacer 2 (ITS2) region was amplified by PCR using the primers described in White et al. (*5*), and the resulting product was sequenced. Sequences were analyzed, and the single nucleotide polymorphism in ITS2 at position 19 (used for differentiation of *B. dermatitidis* from *B. gilchristii*) was noted (*3*). The sequence of 13BL347 ITS2 possessed a cytosine at position 19, which is diagnostic for *B. gilchristii,* whereas thymine at that position is conserved in *B. dermatitidis*. By using a multiple sequence alignment tool, we aligned the 13BL347 ITS2 sequence to sequences of several well-characterized representative sequences of both species (*1*,*3*); it clustered with other *B. gilchristii* strains (Technical Appendix) (*6*).

Variation in clinical presentation among different genetic groups of *B. dermatitidis* has long been observed (*1*–*3*). However, since the 2013 identification of *B. gilchristii*, it is unclear as to whether the previous reports were actually reporting differences in virulence between *B. dermatitidis* and *B. gilchristii*. Therefore, we refer to the fungi that were the subject of previous blastomycosis studies as *Blastomyces* spp. rather than *B. dermatitidis*.

Known virulence factors of *Blastomyces* spp. include the cell surface carbohydrate polymer alpha-1,3-glucan and the *Blastomyces* adhesion 1 protein (formerly WI-1) surface adhesion molecule (*2*). To our knowledge, differences in expression of these virulence factors in *B. dermatitidis* and *B. gilchristii* have not been compared. It has been demonstrated previously that African strains of *B. dermatitidis* differ from North American strains in their growth and morphology and are thought to cause less severe disease (*2*). Genetic analysis has identified 2 distinct genetic groups of African *B. dermatitidis* that differ in expression of the *Blastomyces* adhesion 1 protein surface adhesion molecule (*2*). Whether the differences in growth, morphology, and apparent virulence in the African *Blastomyces* sp. is attributable to *B. gilchristii* and *B. dermatitidis*, intraspecies variation, or a separate undescribed genetic group remains unexplored.

There are several challenges in evaluating the virulence of *B. dermatitidis* and *B. gilchristii*. First, the clinical course of blastomycosis has been found to be correlated with the amount of inoculum (conidia) initially acquired (typically through inhalation) (*7*). In the context of human infection, it is difficult to determine if differences in disease process are attributable to differences in fungal virulence or the magnitude of inoculum. Furthermore, host factors such as immunodeficiency and variation in human leukocyte antigen profile have been shown to cause variation in immune response to *Blastomyces* spp. (*8*).

*Blastomyces* spp. have also been observed to lose virulence during laboratory processing, making the results of previous laboratory-based virulence studies questionable (*7*). Another challenge is that the commercially available molecular probe that is often used to confirm the identification of *Blastomyces* spp. cannot differentiate between *B. dermatitidis* and *B. gilchristii*. Currently, *B. dermatitidis* and *B. gilchristii* cannot be distinguished based on phenotype; PCR followed by sequence analysis is the only method of differentiating these species.

## Conclusions

Most cases of blastomycosis-induced ARDS are preceded by a prodrome of pneumonia weeks to months before development of ARDS (*9*,*10*). However, in a minority of cases, such as the one we describe, the clinical course rapidly progresses to fatal ARDS (*9*,*10*). This patient died within 7 days of hospital admission and <24 hours after intubation. In this case, it is difficult to comment on the virulence of *B. gilchristii* because the patient’s immune status is uncertain. The patient had uncontrolled hyperglycemia secondary to type 1 diabetes mellitus, history of intravenous drug use, and an unknown HIV status; these factors are known to cause immune dysfunction and might have contributed to the acuity and severity of disease. Nevertheless, our findings demonstrate that *B. gilchristii* infection can cause fatal ARDS in humans and form a basis for further virulence and epidemiologic studies of *B. dermatitidis* and *B. gilchristii*.

Technical Appendix Clustal alignment of sequenced region of internal transcribed spacer 2 of *Blastomyces dermatitidis* and *B. gilchristii.*
