# Supplementary material for: Blastomyces gilchristii as Cause of Fatal Acute Respiratory Distress Syndrome
Source: Emerg Infect Dis. 2016 Feb;22(2):306–8. doi: 10.3201/eid2202.151183 (PMC4734522; doi:10.3201/eid2202.151183)
Supplement: Technical Appendix — Clustal alignment of sequenced region of internal transcribed spacer 2 of Blastomyces dermatitidis and B. gilchristii. [file 15-1183-Techapp-s1.pdf]

Article DOI: http://dx.doi.org/10.3201/eid2202.151183

Blastomyces gilchristii as Cause of Fatal Acute Respiratory Distress Syndrome

Technical Appendix

	Strains	1 … 19 … 50
B. gilchristii	TB00029|JN562140	GAAATGCGATAAGTAATG**C**GAATTGCAGAATTCCGTGAATCATCGAATCT
	TB00037|JN562142	GAAATGCGATAAGTAATG**C**GAATTGCAGAATTCCGTGAATCATCGAATCT
	Strain 241|JN562106	GAAATGCGATAAGTAATG**C**GAATTGCAGAATTCCGTGAATCATCGAATCT
	ATCC 66136|JN562118	GAAATGCGATAAGTAATG**C**GAATTGCAGAATTCCGTGAATCATCGAATCT
	ATCC 62583|JN562117	GAAATGCGATAAGTAATG**C**GAATTGCAGAATTCCGTGAATCATCGAATCT
	ATCC 62541|JN562116	GAAATGCGATAAGTAATG**C**GAATTGCAGAATTCCGTGAATCATCGAATCT
	ATCC 60637|JN562115	GAAATGCGATAAGTAATG**C**GAATTGCAGAATTCCGTGAATCATCGAATCT
	ATCC 60636|JN562114	GAAATGCGATAAGTAATG**C**GAATTGCAGAATTCCGTGAATCATCGAATCT
	ATCC-MYA-2585|JN562119	GAAATGCGATAAGTAATG**C**GAATTGCAGAATTCCGTGAATCATCGAATCT
	**13BL347**	GAAATGCGATAAGTAATG**C**GAATTGCAGAATTCCGTGAATCATCGAATCT
B. dermatitidis	ATCC-MYA-2586|JN562161	GAAATGCGATAAGTAATG**T**GAATTGCAGAATTCCGTGAATCATCGAATCT
	ATCC 26197|JN562158	GAAATGCGATAAGTAATG**T**GAATTGCAGAATTCCGTGAATCATCGAATCT
	ATCC 26199|JN562159	GAAATGCGATAAGTAATG**T**GAATTGCAGAATTCCGTGAATCATCGAATCT
	ATCC 28306|JN562159	GAAATGCGATAAGTAATG**T**GAATTGCAGAATTCCGTGAATCATCGAATCT
	UAMH 5635|JN562183	GAAATGCGATAAGTAATG**T**GAATTGCAGAATTCCGTGAATCATCGAATCT
	TB00014|JN562146	GAAATGCGATAAGTAATG**T**GAATTGCAGAATTCCGTGAATCATCGAATCT
	TB00042|JN562148	GAAATGCGATAAGTAATG**T**GAATTGCAGAATTCCGTGAATCATCGAATCT
		****************** *******************************

Technical Appendix Figure. Clustal alignment of sequenced region of internal transcribed spacer 2 (ITS2) of *Blastomyces dermatitidis* and *B. gilchristii.* The ITS2 sequence of isolate 13BL347 was aligned to the ITS2 sequences of several well-characterized representatives of *B. dermatitidis* and *B. gilchristii* sequences (*1*,*2*), using the multiple sequence alignment tool, Clustal Omega (http://www.ebi.ac.uk/tools/msa/clustalo) using default settings (*3*). The single nucleotide polymorphism in ITS2 at position 19 is used for differentiation of *B. dermatitidis* from *B. gilchristii* (*2*). The sequence of 13BL347 ITS2 (underlined) possessed a cytosine at position 19 (bolded white text), which is diagnostic for *B. gilchristii,* while thymine (bolded black text) at that position is conserved in *B. dermatitidis*. Strains included in the alignment were *B. dermatitidis* strains ATCC MYA-2586 (JN562161), ATCC 26197 (JN562158), ATCC 26199 (JN562159), ATCC 28306 (JN562159), UAMH 5635 (JN562183), TB00014 (JN562146), TB00042 (JN562148) (*2*) and alpacaN13BD13_0325 (KF543873) (*1*) and *B. gilchristii* strains ATCC MYA-2585 (JN562119), ATCC 60636 (JN562114), ATCC 60637 (JN562115), ATCC 62541 (JN562116), ATCC 62583 (JN562117), ATCC 66136 (JN562118), strain 241 (JN562106), TB0029 (JN562140), TB0037 (JN562142) (*2*). With the exception of KF543873, these strains were previously identified to species level by Brown et al. (*2*) through multilocus sequence typing scheme using *chs2, drk1, fads, pyrF, tub1, arf6,* and ITS2. *Denotes a conserved nucleotide among strains in alignment.

References

1. Meece JK, Anderson JL, Gruszka S, Sloss BL, Sullivan B, Reed KD. Variation in clinical phenotype of human infection among genetic groups of *Blastomyces dermatitidis.* J Infect Dis. 2013;207:814–22. PubMed http://dx.doi.org/10.1093/infdis/jis756

2. Brown EM, McTaggart LR, Zhang SX, Low DE, Stevens DA, Richardson SE. Phylogenetic analysis reveals a cryptic species *Blastomyces gilchristii*, sp. nov. within the human pathogenic fungus *Blastomyces dermatitidis.* PLoS One. 2013;8:e59237. PubMed http://dx.doi.org/10.1371/journal.pone.0059237

3. Sievers F, Wilm A, Dineen D, Gibson TJ, Karplus K, Li W, et al. Fast, scalable generation of high-quality protein multiple sequence alignments using Clustal Omega. Mol Syst Biol. 2011;7:539. PubMed http://dx.doi.org/10.1038/msb.2011.75
